# Evaluation of Infection and Effective Factors in Impacted Mandibular Third Molar Surgeries: A Cross-Sectional Study

**DOI:** 10.1155/2022/8934184

**Published:** 2022-05-04

**Authors:** Farrokh Farhadi, Parya Emamverdizadeh, Mahdi Hadilou, Parisa Jalali

**Affiliations:** ^1^Department of Oral and Maxillofacial Surgery, Faculty of Dentistry, Tabriz University of Medical Sciences, Tabriz, Iran; ^2^Department of Oral and Maxillofacial Pathology, Faculty of Dentistry, Tabriz University of Medical Sciences, Tabriz, Iran; ^3^Faculty of Dentistry, Tabriz University of Medical Sciences, Tabriz, Iran

## Abstract

**Materials and Methods:**

This cross-sectional study was performed on 148 patients. The incidence of infection was recorded one week, two weeks, and one month after surgery. Independent variables included age, gender, the presence of periapical radiolucent lesions measuring ≥3 mm, the caries of the adjacent second molar, education level of the surgeon, difficulty index, suture type, and flap type. The outcome variable was the incidence of infection.

**Results:**

Among the study subjects, 37.2% were male and 62.8% were female. The mean age was 25.24 ± 4.31 years at the time of surgery. Although early onset infection occurred in 3.4% of patients, none of them developed a delayed-onset infection during the four weeks after surgery. Pederson difficulty index (OR = 3.81, *P*=0.03) and the education level of the surgeon (OR = 0.14, *P*=0.021) were associated with postop infection.

**Conclusions:**

Based on the results of the current study, postop infection was rare. Furthermore, both the Pederson difficulty index and the education level of the surgeon could predict the risk of infection after impacted mandibular third molar surgeries.

## 1. Introduction

Extraction of the impacted third molar is a common outpatient surgery performed in dental clinics [1]. Although it is mostly performed without complications similar to other surgeries, sometimes it might have subsequent complications. The most important complications include alveolitis, pain, dry socket, swelling, dentoalveolar fracture, hemorrhage, suture dehiscence, and the paresthesia of the inferior alveolar nerve [2, 3].

Postsurgery infection is one of the main concerns for most surgeons during impacted tooth surgeries. This complication may occur due to diverse reasons, including systemic diseases, poor surgical techniques, inadequate irrigation of the surgical site, and poor sterilization of instruments [4, 5]. Most infections have some common signs, such as pain, fever, swelling, trismus, clear pus discharge, abscess, and cellulite at the surgical site. However, signs and symptoms, namely, swelling and pain, might also be a normal tissue response after surgery and not a definite symptom of infection [6, 7]. Although an accurate diagnosis of infection is difficult, the onset of infection can be defined by the presence of purulent discharge at the surgical site and painful induration [8].

Various factors are involved in the onset and severity of infection, such as general health condition of the patient, systemic diseases, age, gender, dental anatomy, difficulty index, degree of impaction, surgeon's experience, surgical technique, flap type, and presence of periapical radiolucent lesions measuring ≥3 mm [3, 8, 9]. Infection following a wisdom tooth extraction is most common in the lower rather than the upper jaw. Moreover, the infection rate after impacted third molar surgeries is greater than routine tooth extraction [7]. Some previous studies reported infection rates of 1.5%–5.8% and 0.9%–4.3%. Approximately 50% of infections are localized subperiosteal abscesses that occur 2–4 weeks after surgery, usually due to the debris remaining under the mucoperiosteal flap, and can be easily removed by surgical debridement and drainage [3, 6].

Many efforts have been made to investigate the incidence of postoperative infection and its associated risk factors. The rate of impacted wisdom teeth surgeries is high, and preventive measures to reduce postoperative infection are of importance. Therefore, the present study aimed to evaluate the incidence of infection and the factors affecting it in hard tissue impacted mandibular third molar surgeries. The statistical null hypothesis of the study was the lack of relationship between the predictor variables and the risk of infection.

## 2. Materials and Methods

This cross-sectional study was performed on 148 patients who referred to the Dentistry Faculty Clinic of Tabriz University of Medical Sciences, Iran, during September 2020–May 2021. The study protocol was approved by the Research Ethics Committee of Tabriz University of Medical Sciences (IR.TBZMED.REC.1399.610). Informed consent was obtained from all patients. All eligible patients referring for hard tissue impacted third molar surgery were included in the study. The inclusion criteria entailed eagerness to participate in the study, age over 18 years, no systemic diseases, no acute inflammation in the area of surgery, and following the given oral hygiene education. The exclusion criteria included having systemic diseases, acute or chronic periapical inflammation, need for prophylaxis, and certain complications during surgery, such as the manipulation of the inferior alveolar canal and inability to close the flap completely with sutures or developing a dry socket.

After obtaining informed consent from all patients, data were collected using a researcher-made checklist. Panoramic radiographs and, if necessary, cone-beam computed tomography images were taken from all participants before surgery. The surgeries were performed by oral and maxillofacial surgeons or residents. All instruments used in the surgeries were sterilized in the Central Sterilization Unit of Tabriz Dental School. The surgeries were performed utilizing a low-speed handpiece with abundant physiologic serum irrigation in accordance with the principles of infection control. After the surgery, antibiotics, including amoxicillin 500 mg (every 8 hours for 7 days), and analgesics, such as Gelofen 400 mg and acetaminophen 325 mg (every 6 hours), were prescribed. Clindamycin 150 mg was administered for patients with hypersensitivity to amoxicillin.

Patients were referred for check-ups during the first week, the second week, and one month postsurgery, and the existing of infections was recorded. The predictor variables investigated in this study encompassed age, gender, presence of periapical radiolucent lesions measuring ≥3 mm, caries of the adjacent second molar, education level of the surgeon (resident or maxillofacial surgeon), Pederson difficulty index (easy (3-4), medium (5–7), and hard (8–10)) [10–12], suture type in terms of absorbability and nonabsorbability, flap type (triangular or envelop), and clinical examination of early onset infection (EOI) and delayed-onset infection (DOI) signs and symptoms. The EOI was defined as an infection occurring during the first week and DOI as an infection occurring after 7 days of surgery. The appearance of infection was defined by purulent discharge and painful induration.

### 2.1. Statistical Analysis

Data were statistically analyzed using the SPSS software version 17 (SPSS Inc., Chicago, IL, USA). Values were presented as means, standard deviations, and frequencies/percentages. The normal distribution of data was evaluated by the Kolmogorov–Smirnov test. To investigate the relationships between dependent and independent variables, the Mann–Whitney *U* test and Pearson's chi-square test were used. Moreover, the logistic regression model was applied to investigate the relationships and intensity of the predictor factors on the incidence of infection. The significance level was considered *P* < 0.05.

## 3. Results

The age range of patients was 18–35 years, with a mean of 25.24 ± 4.31 years. We observed that 55 (37.2%) cases were male and 93 (62.8%) were female. None of the patients quit the study due to complications. The EOI occurred in 5 (3.4%) participants, while no DOI cases were observed. Four weeks after the surgery, no periosteal infection was found, and all patients had a normal wound healing process.

A comparison of the distribution of predictor variables concerning postoperative infection is given in Table 1. The mean age of patients who developed postoperative infection was similar to those who did not have an infection (*P*=0.996). The postoperative infection was not associated with gender (*P*=0.651), flap type (*P*=0.584), suture type (*P* > 0.999), periapical radiolucent lesions measuring ≥3 mm (*P*=0.558), adjacent second molar caries (*P*=0.782), and the side of the mandible on which surgery was performed (*P* > 0.999). However, postoperative infection was associated with difficulty index (*P*=0.013) and the education level of the surgeon (*P*=0.028) (Table 1). Furthermore, these associations were confirmed by the logistic regression model (Table 2). In other words, the incidence of infection increased 3.81 times with the level of difficulty index (OR = 3.81, *P*=0.03) and decreased by 86% with higher education level of the surgeons (OR = 0.14, *P*=0.021).

## 4. Discussion

Infection control and prevention is one of the most vital steps in surgeries. Therefore, this study aimed to evaluate infection incidence after mandibular impacted third molar surgeries and its risk factors in the Iranian population. The results of the present study showed that the education level of the surgeon is a risk factor for infection incidence. Therefore, the null hypothesis was rejected. The incidence of infection decreased by 86% with each higher education level of the surgeons (OR = 0.14, *P*=0.021), which is in line with the findings of the previous studies [8, 13–15]. Education level cannot completely reflect the experience of a surgeon, which can perfectly be measured by the number of surgeries a surgeon has performed. However, the educational degree could represent a part of a surgeon's experience because the measurement of experience could be quite difficult.

In the present study, the prevalence of infection was 3.4%, which was in the same range reported in the previous investigations (0.4%–6%) [9, 13, 16, 17]. The postoperative infection rate of mandibular third molar surgery was reported as 1.94% in a study with a similar methodology by Sukegawa et al. [9]. The greater rate of infection in the current study could be due to the different education levels of the surgeons. Specialists had performed surgeries in the research by Sukegawa et al., whereas both specialists and 1^st^ to 3^rd^ year residents performed surgeries in the present study. Brunello et al. [13] reported that the DOI rate was 3.7% after mandibular third molar surgery. They performed surgeries on partial and nonretention mandibular third molars, as well as the completely impacted ones. Their results are almost close to the current study, mostly due to having both specialists and residents as surgeons.

There are some contradictory reports in the literature regarding the diverse rates of postoperative complications in males and females. For example, while Blondeau et al. [8] reported a greater infection rate in females and Muhonen et al. [18] revealed a higher infection rate in males. However, in the present study, no relationship was found between gender and infection (*P*=0.651).

Difficulty index was one of the predictor factors associated with an increased risk of postoperative infection. Previous studies reported bone removal and tooth section as risk factors for complications after latent molar surgery. Surgeries on the impacted teeth are much more difficult than other operations and require more time and a wider flap design. Therefore, postoperative complications are more likely in these surgeries [19].

It is important to note that although the principal protocols for infection control in surgery are the same, there are variations in countries. This can affect the incidence of infection. Studies have shown that the difficulty index is associated with the difficulty of surgery and the duration of operation [20]. The current study also demonstrated that the difficulty index is useful for predicting the probability of developing a postoperative infection after mandibular impacted third molar surgeries. The incidence of infection rose 3.81 times with a higher level of difficulty index (OR = 3.81, *P*=0.03).

In a retrospective study on 178 mandibular molars, Figueiredo et al. [21] stated that lower third molar teeth with complete soft tissue retention, distal space deficiency, and vertical or mesioangular tilt probably lead to DOI. Furthermore, Sukegawa et al. [9] indicated that the depth of impaction is a risk factor for infection after surgery. In the present study, in order to determine the difficulty index, Pederson's classification was used, which is the combination of Winter, Pell, and Gregory's classifications [10–12]. Based on the results of this study, although the risk of infection was not associated with distal space, depth of impaction, and angulation factors separately, it was associated with difficulty index as a combination of them. This shows the thoroughness of Pederson difficulty index.

The rate of infection reported in our study is almost identical to other investigations. However, it is noteworthy that some studies included different types of tooth occlusion, namely, hard and soft tissue occlusions. Some investigations have reported the incidence of infection per individuals and others per teeth. Moreover, sample size can also be effective in disease incidence because a large number of cases lead to a significant number of complications.

### 4.1. Strengths and Limitations

The major strength of this study was assessing infection occurrence by observing the site of surgery in the follow-ups. Consequently, the outcome measurement method was independent of patients' answering (recall bias). However, the cross-sectional design of the current research could be considered a limitation due to the lower capability of demonstrating causality between variables. Therefore, it is suggested to conduct randomized controlled trials with solid designs for future studies to reach more robust and reliable conclusions.

## 5. Conclusion

According to the results of this study, both the difficulty index obtained by Pederson's classification and the education level of the surgeon were appropriate predictors of the risk of postoperative infection in the impacted third molar surgeries. Although infection after mandibular third molar surgeries is rare, surgeons and dentists should consider the possibility of this complication and follow patients up for at least one month after surgeries.

## Figures and Tables

**Table 1:** Association between the predictor variables and the outcome variables.

Variables		Postoperative infection	No postoperative infection	*P* value
Total		5	143	
Age		25 ± 1.20	25 ± 1.35	0.996

Gender	Male	1	54	0.651
	Female	4	89	

Position of tooth	Right	3	89	>0.999
	Left	2	54	

Distal space	I	0	54	0.096
	II	3	71	
	III	2	18	

Depth of impaction	A	0	28	0.087
	B	1	68	
	C	4	47	

Angulation	Horizontal	2	45	0.763
	Vertical	3	71	
	Distoangular	0	4	
	Mesioangular	0	23	

Pederson difficulty index	Easy (3–4)	0	16	0.013^*∗*^
	Medium (5–7)	1	95	
	Hard (8–10)	4	32	

Periapical radiolucent lesions	Presence	1	21	0.558
	Absence	4	122	

Adjacent second molar caries	Presence	1	45	0.782
	Absence	4	94	
	No adjacent tooth	0	4	

Surgeon's education level	1^st^ year resident	3	19	0.028^*∗*^
	2^nd^ year resident	2	69	
	3^rd^ year resident	0	41	
	Maxillofacial surgeon	0	14	

Type of flap	Envelope	5	115	0.584
	Triangular	0	28	

Type of suture	Nonabsorbable	5	121	>0.999
	Absorbable	0	22	

^*∗*^Statistically significant.

**Table 2:** Logistic regression analysis of predictors of inflation occurrence.

Variables	OR	SE	*P* value
Difficulty index	3.81	0.616	0.030^*∗*^
Surgeon's education level	0.14	0.834	0.021^*∗*^
Distal space	4.34	0.736	0.46
Depth of impaction	6.61	1.061	0.075
Angulation	0.68	0.930	0.689

^*∗*^Statistically significant. OR, odds ratio; SE, standard error.

## Data Availability

The data used to support the findings of this study are available from the corresponding author upon request.
